# The Role of Ovarian Sex Steroids in Metabolic Homeostasis, Obesity, and Postmenopausal Breast Cancer: Molecular Mechanisms and Therapeutic Implications

**DOI:** 10.1155/2015/140196

**Published:** 2015-03-19

**Authors:** Viroj Boonyaratanakornkit, Prangwan Pateetin

**Affiliations:** ^1^Department of Clinical Chemistry, Faculty of Allied Health Sciences, Chulalongkorn University, 154 Rama I Road Patumwan, Bangkok 10330, Thailand; ^2^Graduate Program in Clinical Biochemistry and Molecular Medicine, Thailand

## Abstract

Obese postmenopausal women have an increased risk of breast cancer and are likely to have a worse prognosis than nonobese postmenopausal women. The cessation of ovarian function after menopause results in withdrawal of ovarian sex steroid hormones, estrogen, and progesterone. Accumulating evidence suggests that the withdrawal of estrogen and progesterone causes homeostasis imbalances, including decreases in insulin sensitivity and leptin secretion and changes in glucose and lipid metabolism, resulting in a total reduction in energy expenditure. Together with a decrease in physical activity and consumption of a high fat diet, these factors significantly contribute to obesity in postmenopausal women. Obesity may contribute to breast cancer development through several mechanisms. Obesity causes localized inflammation, an increase in local estrogen production, and changes in cellular metabolism. In addition, obese women have a higher risk of insulin insensitivity, and an increase in insulin and other growth factor secretion. In this review, we describe our current understanding of the molecular actions of estrogen and progesterone and their contributions to cellular metabolism, obesity, inflammation, and postmenopausal breast cancer. We also discuss how modifications of estrogen and progesterone actions might be used as a therapeutic approach for obesity and postmenopausal breast cancer.

## 1. Introduction

The prevalence of obesity in many developed and developing countries has been increasing at an alarming rate reaching pandemic proportions over the past decade [1]. By the year 2030, the number of overweight and obese adults is projected to be 1.35 billion and 573 million individuals, respectively, worldwide [2]. A recent study estimated that one in five deaths in the United States is associated with obesity, surpassing smoking as Americans' number one killer [3]. Health problems that can be attributed to obesity include type 2 diabetes, cardiovascular diseases, hypertension, and cancer of several organs. While the relationship between obesity, diabetes, and cardiovascular diseases has been well studied and documented, the relationship between obesity and cancer has only started to receive much attention in recent years (see review [4]).

An increasing number of studies have highlighted the association between obesity and the risk of various cancers. It is estimated that 15–20% of all cancer deaths in the United States can be ascribed to obesity [5]. A recent study estimated that for every 5 kg/m^2^ increase in body mass index (BMI), a risk for developing esophageal, thyroid, and colon cancer in males increased by 52%, 33%, and 24%, respectively, whereas the risk for developing endometrial and postmenopausal breast cancer in females increased by 59% and 12%, respectively [6]. Interestingly, the association between obesity and postmenopausal breast cancer was found to be highest in women in the Asia-Pacific region with a 31% increase in postmenopausal breast cancer risk for every additional 5 kg/m^2^ increase in BMI [6]. Epidemiological data suggests that women with breast cancer who are obese at the time of their diagnosis are more likely to have a worse prognosis than nonobese, lean women [7]. Prospective cohort studies showed about a twofold increase in breast cancer risk among postmenopausal women who had higher production of various sex steroid, including dehydroepiandrosterone sulfate (DHEAS), testosterone, estrone, and total estradiol (E2) and breast cancer risk was inversely correlated with the expression of steroid hormone binding globulin (SHBG) [8]. Analysis of several cohort studies indicated that the association of high BMI with increased breast cancer risk could be attributed mostly to elevated bioavailable E2 [9]. Mortality was also higher among obese women with breast cancer than leaner women [10]. Meta-analysis of 43 studies of comorbidity of obesity and breast cancer revealed that obese patients were 33% more likely to die from breast cancer than nonobese patients. Recent evidence suggested that metabolic syndromes such as insulin resistance, hypertension, and hyperlipidemia increased the risk for postmenopausal breast cancer [11], suggesting a central role of ovarian sex steroids, estrogen, and progesterone, in regulating cellular metabolism, proliferation, and differentiation.

Obesity causes dysregulated metabolism and also provokes chronic inflammation in adipose tissue [12, 13]. It is now widely accepted that cancer is involved in the alteration of cellular metabolism [14] and inflammation [15]. Ovarian sex steroids, estrogen, and progesterone and their cognate receptors (estrogen receptor (ER) and progesterone receptor (PR)) have also been shown to influence metabolism and inflammatory responses. Loss of the ovarian function to supply estrogen and progesterone after menopause can cause deregulation of the body's metabolism and inflammatory responses with increased risk of postmenopausal breast cancer. In this review, we will discuss and provide an integrated view of our current understanding of this complex relationship between ovarian sex steroids and their receptors in relation to obesity and inflammation and their contribution to postmenopausal breast cancer. Detailed molecular mechanisms of how ovarian sex steroids affect obesity and inflammation will also be discussed.

## 2. Cancer and Metabolism

Cancer is often associated with alterations in cellular metabolism. In 1920, Warburg found that cancer cells prefer to metabolize glucose by glycolysis as compared to oxidative phosphorylation even in the presence of ample oxygen [16]. While the Warburg effect is less efficient in producing ATP it is very effective in providing cellular building blocks and macromolecules such as amino acids, lipids, and nucleic acids [17], allowing cancer cells to rapidly proliferate. For example, pyruvate kinase (PK), a rate-limiting step enzyme responsible for changing phoshphoenolypruvate (PEP) to pyruvate, is expressed in most cell types as four different isoforms PKL, PKR, PKM1, and PKM2. Embryo or stem cells express only PKM1 while proliferating and cancer cells express mainly PKM2 [18]. PKM2 is less effective in converting PEP into pyruvate resulting in a shortage of pyruvate available for the tricarboxylic acid (TCA) cycle, and oxidative phosphorylation, making a more favorable metabolic environment for glycolysis. Inhibition of PKM2 expression by short-hairpin RNA (shRNA) resulted in an increase in oxygen consumption and a decrease in pyruvate production [19].

A number of studies have suggested that tumor microenvironment promotes changes in cellular metabolism. Several factors have been shown to shift cellular metabolism toward aerobic glycolysis including ovarian sex steroid signaling, cellular microenvironment, obesity, and inflammation [20–23]. Thus, the combined effect of menopause, obesity, and inflammation could create the right environment to foster development and progression of breast cancer. How these factors alter metabolic pathways and cellular metabolism will be discussed below.

## 3. Menopause and Obesity

### 3.1. Role of Estrogen and ER Signaling in Metabolic Control and Homeostasis

Postmenopausal women are more prone to gain weight. However, it is unclear why or how the menopausal transition leads to weight gain and increased breast cancer risk. The physiological withdrawal of ovarian sex steroids, estrogen, and progesterone has been implicated in altered metabolism after menopause. Estrogens have been shown to play an important role in metabolic control and homeostasis. There are three forms of estrogen in women, estrone (E1), estradiol (E2), and estriol (E3). The major and most potent circulating estrogen in women throughout the reproductive years is E2. A key enzyme that aromatizes androgen into estrogen and is required for E2 biosynthesis is a member of P450 enzyme family, aromatase [24]. Meta-analysis of several clinical studies revealed a connection between E2 and control of several key metabolic functions including abdominal obesity, insulin sensitivity, lipid transport, blood pressure, and inflammatory or prothrombotic states [25].

### 3.2. Role of E2/ER in Insulin-Sensitivity and Glucose Uptake

E2 and estrogen receptors (ER*α* and ER*β*) are involved in blood glucose and lipid homeostasis [26]. ER*α* knockout (ER*α*KO) mice as well as aromatase knockout (ArKO) mice are obese and insulin resistant [27, 28]. Mutation of the aromatase gene or males with genetic ER*α* deficiency developed insulin resistance and glucose intolerance [29, 30]. Microarray analysis revealed that genes involved in hepatic and lipid biosynthesis were upregulated while genes involved in lipid transport were decreased in ER*α*KO mice. Interestingly, ER*β* knockout (ER*β*KO) mice showed normal glucose tolerance and insulin release, suggesting that ER*β* at most plays a minor role in regulating body's metabolism [31]. Deletion of ER*α* gene in mouse myeloid cells showed adipose tissue inflammation with insulin resistance, acceleration of atherosclerotic lesion, and obesity [32]. Several lines of evidence indicate that ER*α* is the major form of ER that plays a predominant role in regulating glucose and lipid metabolism [27, 32, 33]. ER*α* plays a central role in regulating metabolism and physical activities in the brain [34, 35]. Mice lacking ER*α* in the hypothalamic steroidogenic factor 1 neuron showed a significant decrease in energy expenditure and an increase in abdominal fat [35]. Ovariectomized (OVX) mice fed with high fat diet showed increased adiposity and insulin insensitivity, and E2 treatment improved insulin sensitivity and reduced liver fat deposit. However, E2 failed to improve insulin resistance and fatty liver induced by high-fat diet in hepatocyte-specific ER*α*KO mice [33]. In addition, ER*α* could help induce insulin sensitivity through GLUT4, a major insulin-stimulated glucose transporter in muscle cells and adipocytes. In aging female rats, E2 treatment increased insulin sensitivity and improved glucose homeostasis through increasing GLUT4 expression in muscle cells [36]. The E2 increase of GLUT4 expression is likely mediated through ER*α* since ER*α*KO mice are glucose intolerant and insulin resistant [28]. On the other hand, both glucose tolerance and insulin sensitivity in ER*β*KO mice are normal or better than their wild-type littermates [31]. Together, these data suggest that E2 through ER*α* regulates insulin sensitivity and lipid biosynthesis and transport.

How E2 and ER*α* mediate an increase in insulin sensitivity and decrease adiposity is likely to be multifactorial. ERs are expressed in all metabolically important tissues including brain, adipose tissue, skeletal muscle, and pancreas [37]. E2 suppresses accumulation of white adipose tissue (WAT) by decreasing fatty acid and triglyceride synthesis. OVX female mice treated with E2 showed a decrease in adipocyte size by reducing fatty acid uptake through decreasing lipoprotein lipase expression, by reducing lipogenesis through decreasing acetyl-coA carboxylate and fatty acid synthase expression, and by increasing lipolysis through catecholamine activation [38].

### 3.3. Role of E2/ER*α* in Cellular Metabolism of Glucose and Lipid Metabolism

At the cellular level, E2 and ER*α* have been shown to directly regulate glucose and lipid metabolism. E2/ER*α* affects several key processes of glucose metabolism including glycolysis, the TCA cycle, and oxidative phosphorylation [39]. E2 increases the expression and activities of several key glycolytic enzymes. Glycolysis in MCF-7 breast cancer cells was shown to be induced by E2 and inhibited by tamoxifen (TAM) [40]. E2 induced several key glycolytic enzymes in the cytosol such as hexokinase (HK), phosphofructokinase (PFK), and pyruvate kinase (PK) in the female rat brain after 4 hours of treatment [41]. Proteomic analysis of E2-regulated proteins in the bones of female mice found that both glycolytic enzymes, enolase and pyruvate kinase isoform M2 (PKM2), were regulated by E2 [42]. In addition, E2/ER*α* also regulated several key enzymes of the TCA cycle found in the inner membrane of the mitochondria. Treatment of OVX rats with E2 increased the activity of citrate synthase, a key enzyme condensing an acetyl group from acetyl-CoA with oxaloacetate to form citrate in TCA cycle, in muscle cells and in cerebral blood vessel cells [43, 44]. Proteomic analysis of brain mitochondrial proteins after treatment of OVX rats with E2 for 24 hours showed significant increased expression of aconitase, pyruvate dehydrogenase isoform E1b and E2, and malate dehydrogenase, as well as enzymes coupling TCA with amino acid synthesis such as 2-oxoglutarate dehydrogenase and glutamine dehydrogenase [45]. These data underscore the significance of E2/ER in regulating glucose metabolism in glycolysis and the TCA cycle. Local production of E2 after menopause could create a microenvironment conducive to aerobic glycolysis metabolism to promote cell proliferation and cancer development.

Oxidative phosphorylation occurs in the mitochondrial inner membrane and generates the majority of cellular energy in the form of ATP, NADH, and FADH [39]. Studies have shown the significance of ERs in mitochondria and oxidative phosphorylation. Both forms of ERs, ER*α* and ER*β*, have been shown to localize to the mitochondria in several E2-target cells including uterine cells, neurons, cardiomyocytes, bone cells, hypothalamic cells, endothelial cells, liver cells, and breast cancer cells [46–50]. Thirteen proteins in the mitochondrial respiratory chain complexes are encoded by the mitochondrial DNA (mtDNA) within the mitochondria, whereas the majority of proteins required for mtDNA replication, transcription, and translation are encoded by nuclear DNA. Several studies in various cell types have demonstrated that E2 stimulated mtDNA transcription [44, 48, 51, 52], and ICI182780, a pure ER antagonist, inhibited E2-induced mtRNA gene expression [48], demonstrating the significance of E2 and mitochondrial ER in regulating mtDNA gene transcription. E2 not only activated mtDNA gene transcription, but also induced expression of nuclear DNA of genes encoding MRC proteins leading to E2-induced mitochondrial respiration [39]. Together, these data suggest that E2/ER coordinately induces transcription of mtDNA and nDNA encoding respiratory chain subunits and other regulatory proteins required for mitochondria replication and function. Therefore, E2 withdrawal or defects in ER functions could result in a decrease in mitochondrial respiration and a reduction in energy expenditure. In fact, OVX-induced obesity in mice was associated with decreased oxygen consumption, indicating a reduction in energy expenditure in the absence of E2 [21]. Similar reduction in energy expenditure in the mitochondria could also be observed in women after menopause [53].

E2 also plays an important role in fatty acid *β*-oxidation. Analysis of liver collected from aromatase knockout mice with undetectable levels of E2 revealed a reduction of mRNA expression and activity of enzymes involved in fatty acid metabolism. E2 treatment of aromatase knockout mice restored mRNA expression and increased activity of fatty acid metabolism enzymes [54]. Analysis of adipose tissue and muscle from female mice at 2–4 weeks after ovariectomy showed that nuclear receptors and proteins required for efficient energy expenditure such as PPAR*γ*, PPAR*δ*, PCG1*α*, PCG1*β*, and ERR1 were all reduced as compared to sham-operated control mice [55]. In addition, expression of enzymes involved in fatty acid *β*-oxidation and transcription factors required for lipolysis were also decreased [55]. Thus, depletion of E2 by ovariectomy or during menopause may lead to a decrease in expression of genes required for efficient energy expenditure and genes for fatty acid or lipid catabolism which may contribute, in part, to obesity after menopause.

### 3.4. Role of E2/ER in Adipokine Signaling

In addition to directly affecting adipocyte metabolism, E2/ER signaling interacts with adipokine, leptin, and adiponectin signaling [56]. Leptin regulates energy intake and expenditure, and leptin levels are directly proportional to the amount of adipose tissue in the body. Leptin binds to leptin receptors in the hypothalamus and inhibits appetite and food intake (see review [57]). Studies have shown that E2 may mediate antiobesity effects through ER*α* in the hypothalamus, in the ventral medial nucleus (VL VMN), and the arcuate (ARC), by increasing expression of leptin receptors, thus increasing leptin sensitivity when E2 levels are higher [58]. Leptin levels correlate with the levels of estrogen in premenopausal women; however, this correlation between leptin and estrogen often disappears in postmenopausal women and could help explain a rise in obesity in the postmenopausal woman population [59].

Adiponectin is an important adipokine that is produced by adipose tissue and regulates peripheral glucose and insulin levels [60]. Studies suggest that ER*α* is a positive regulator of adiponectin levels in adipose tissue. After menopause, there is a shift in the balance between ER*α* and ER*β* in adipose tissue, with ER*β* becoming more dominant [61]. Secretion of adiponectin from adipose tissue is stimulated by peroxisome proliferator activated receptor gamma (PPAR*γ*). ER*β* has been shown to reduce PPAR*γ*-expression and activity resulting in a reduction in adiponectin secretion from adipose tissue [62] and may result in decreased insulin levels associated with menopause. More studies will be needed to determine the role of adiponectin in metabolic disorder such as menopause-induced obesity.

Therefore, E2 withdrawal in postmenopausal women could lead to a decrease in insulin and leptin sensitivity and a decrease in mitochondria oxidative phosphorylation and lipid metabolism. These changes in glucose and lipid uptakes along with a decrease in carbohydrate and lipid metabolism render postmenopausal women less efficient in energy expenditure and more susceptible to obesity after E2 withdrawal.

## 4. Molecular Mechanism of Metabolic Control by ER***α***: Master Regulator of Glucose and Lipid Metabolism

Different forms of ERs mediate distinct functions of E2. Recent data strongly indicates that the beneficial effects of E2 on metabolic regulation are mediated through ER*α*. ER*α* is a member of the nuclear receptor superfamily and comprises six modular domains including the N-terminal domain or the A/B domain, DNA binding domain, hinge region, ligand binding domain, and the C-terminal or the E domain [63, 64]. ER*α* contains two activation functions (AFs) with AF-1 in the A/B domain and AF-2 in the ligand binding domain [65]. ER*α* can mediate its biological effects through nuclear and extranuclear signaling [63]. In nuclear signaling, ER*α* directly binds to estrogen response elements (ERE) or interacts with other transcription factors to regulate gene transcription in the nucleus. In extranuclear signaling, ER*α* interacts with cytoplasmic signaling molecules or other signal transduction scaffolding proteins and activates many key cytoplasmic signaling pathways (see review [66, 67]). A recent study suggested that the AF2 domain is essential for E2-mediated control of glucose homeostasis [68]. Mice expressing a mutant ER*α* lacking the AF2 domain rapidly gained weight and became severely obese with insulin resistance and glucose intolerance symptoms, similar to ER*α* knockout mice, whereas mice expressing a mutant ER*α* lacking the AF1 domain had similar body weight and metabolic functions to wild-type mice. E2 treatment in mice with wild type or AF-1-deleted ER*α* showed increased energy expenditure and insulin sensitivity but failed to show these beneficial metabolic effects in mice with AF-2-deleted ER*α* [68].

However, detailed molecular mechanisms of how ER*α* mediated its beneficial metabolic effects are not well understood. Mice expressing the ER*α* mutant with disrupted ERE binding showed a decrease in body weight, improved insulin sensitivity, and increased energy expenditure after E2 treatment. These data suggested that ER*α* likely mediated its metabolic effects in an ERE-independent manner, possibly through tethering with other transcription factors such as activator protein-1 (AP-1), specificity protein 1 (Sp1), or nuclear factor *κ*B (NF-*κ*B) [69]. More studies will be required to determine the role of AF2 in E2-mediated ERE-independent signaling of ER*α* in metabolic regulation. ER*α* activation through AF-1 or through both AF-1 and AF-2 has been shown to promote breast cancer proliferation [70]; however, only AF-2 activation is needed for ER*α*-mediated metabolic regulation. Therefore, it would be of great interest therapeutically to develop an ER*α* AF-2 agonist to harness the beneficial metabolic effects of ER*α* without the harmful effects of ER*α* AF-1 [71].

## 5. Role of Progesterone and PR Signaling in Metabolic Control and Homeostasis

After menopause, cessation of ovarian functions leads to the withdrawal of both estrogen and progesterone. While the role of E2/ER signaling in metabolic controls has been well documented, how progestins and PR signaling influences metabolic control and homeostasis is still unclear. Biological effects of progestin are mediated through PR. PR exits as two isoforms: full-length PR-B and 164-amino acid N-terminal truncated, PR-A [72]. The two isoforms of PR play distinct roles both* in vitro* and* in vivo*. In most cases, PR-B acts as a stronger transcriptional activator as compared to PR-A. PR-A can ligand-dependently act as a transcriptional repressor of ER, androgen receptor (AR), or glucocorticoid receptor (GR) [73]. Selective deletion of the PR isoform showed distinct functions* in vivo* [74, 75]. PR mediates its actions by binding ligands, translocating into the nucleus, and directly binding to DNA or nuclear signaling. In addition, PR can also directly interact with cytoplasmic signaling molecules and activate cytoplasmic signaling pathways such as Src/Ras/Raf/MAPK [76], PI3K/Akt, or Src/Stat3 [77] (see review [78]). Progesterone regulates genes that are involved in RNA and protein processing, cell cycle, apoptosis, and cellular metabolism [79]. Microarray gene expression studies showed that PR regulates several important genes involved in cholesterol or steroid metabolism and trafficking, fatty acid and lipid metabolism, and nucleotide and amino acid metabolism in breast cancer cells [79].

The role of progesterone/PR signaling in metabolic control is not well understood. PRs have been shown to be expressed in adipocytes [80], where they prevent lipolysis in rat adipose tissue [81]. A recent study showed that progesterone inhibits glucose uptake in 3T3-L1 adipocytes through a decrease in IRS-1 expression leading to a decrease in IRS phosphorylation, the association of IRS-1 with p85, and a subsequent decrease in Akt1 and Akt2 phosphorylation [82]. Progesterone activates glycogen phosphorylase (a rate-limiting step in glycogenolysis) or the breakdown of glycogen through an extranuclear signaling pathway independent of cAMP leading to increased blood glucose levels [83]. Rats treated with progesterone for 14 days showed a decrease in GLUT4 in both adipose and skeletal muscle cells [84]. While estrogen appears to increase the capacity of carbohydrate and lipid metabolism, progesterone seems to abolish estrogen-induced metabolic effects in skeletal muscle cells [84]. However, progesterone appears to exert different metabolic influences in cancer cells. In breast cancer cells medroxyprogesterone acetate (MPA) treatment for 6 days increased glucose uptake and fatty acid synthase (FASN) and activation of stearoyl-CoA desaturase-1 (SCD-1). These changes in FASN and SCD-1 activities increased monounsaturated fatty acid production and contributed to an increase in cellular phospholipids, triglycerides, and formation of lipid droplets in breast cancer cells [85].

Effects of progesterone on metabolic control have been extensively studied in gestational diabetes. Progesterone levels increase to support embryo transplantation and inhibit ovulation during pregnancy. Hepatic glucose production increases 15–30% to accommodate fetal needs, which is compensated by a 50–70% decrease in insulin-stimulated glucose uptake in late pregnancy [80]. This decrease in insulin sensitivity or insulin resistance is one of a number of the metabolic changes associated with pregnancy, and 3–7% of pregnant women develop impaired glucose tolerance or diabetes [81]. Pregnancy is an altered metabolic state where there is an increase in the production of insulin from *β*-cells to facilitate an increase in glucose uptake. However, progesterone could also alter insulin insensitivity. Pregnant rats treated with progesterone are more insulin resistant than placebo-treated control rats [82]. Studies suggest that progesterone-induced insulin resistance may be due to reductions in Glut4 expression and glucose uptake in skeletal muscle cells [83]. In C57BL/6 mice, nonfasting plasma glucose is correlated with progesterone levels [86]. High dose progesterone treatment accelerated the progression of diabetes in female obese diabetic-prone db/db mice and treatment with RU486 increased insulin and reduced blood glucose levels in both wild-type and db/db mice [86].

PR knockout mice showed lower fasting blood sugar and improved glucose tolerance compared to wild-type mice due to enhanced insulin secretion caused by an increase in *β*-cell proliferation and *β*-cell mass [86]. Interestingly, PR knockout improved insulin sensitivity only in female mice, not in male mice, suggesting that other factors, other than PR, might play a role in insulin sensitivity in males. A recent study showed that PR activation enhanced the proinflammatory cytokine- PIC-induced cell injury in Min6 mouse pancreatic islet *β*-cells, possibly through an increase in the mitogen-activated protein kinase (MAPK) signaling and p53 expression and a decrease in Akt signaling. A nonsteroidal PR antagonist or PR siRNA interference protected *β*-cells from PIC-induced injury [87]. Together, these data suggest that progestin-mediated activation of PR could have adverse effects on insulin sensitivity possibly through a decrease in insulin secretion. In postmenopausal women, the absence of progesterone is likely to enhance insulin production and could promote insulin resistance. In addition, progesterone via PR has been shown to activate expression of insulin-like growth factor binding protein-1 (IGFBP1) that sequesters and inhibits insulin-like-growth factor-I (IGF-I) action [88, 89]. Physiological withdrawal of progesterone after menopause could make IGF-1 more available to promote cell proliferation. Along with the dysregulated metabolic state induced by estrogen withdrawal, the combination of both estrogen and progesterone withdrawal in postmenopausal women could strongly increase the risk for obesity and breast cancer. Together, these data suggest the interplay of obesity, ER PR expression, and breast cancer aggressiveness. However, more studies will be required to examine detailed molecular mechanisms of these interactions. Better understanding of these interactions will help us to develop better and more effective treatments for postmenopausal breast cancer.

## 6. Obesity and Inflammation Increase Risk for Postmenopausal Breast Cancer

**Figure 1:**
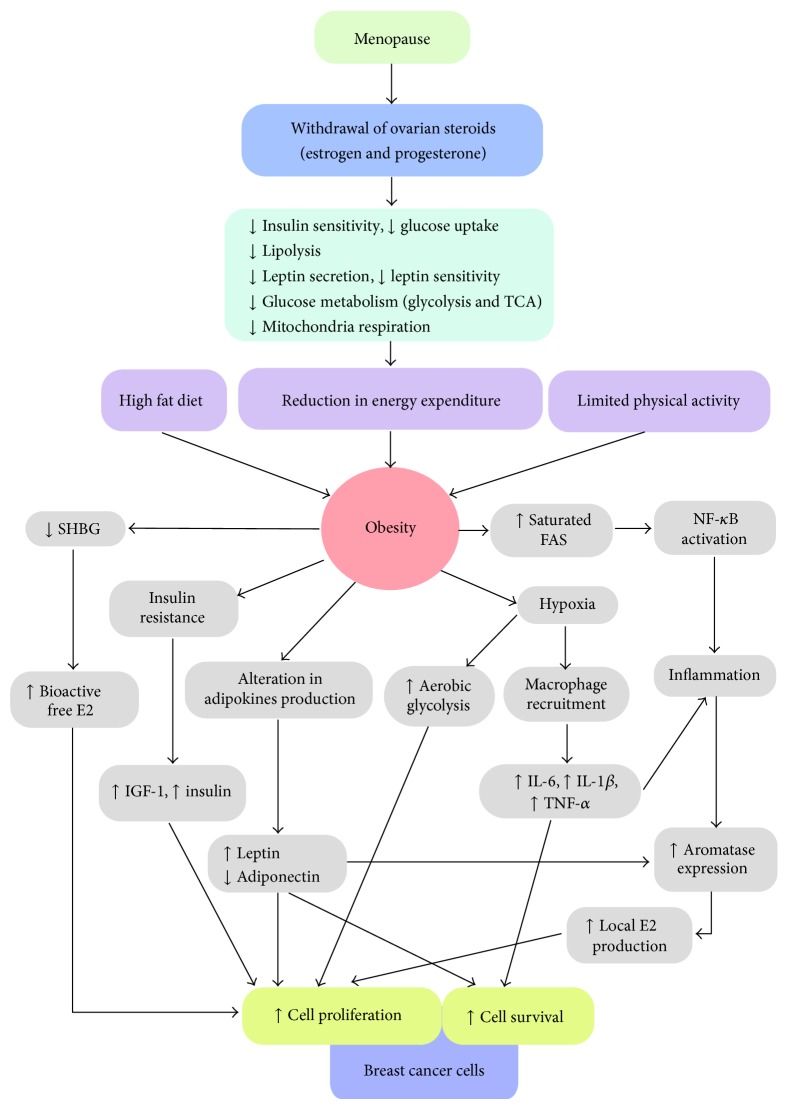
Diagram linking menopause and alterations in cellular metabolism with obesity and breast cancer. FAS: saturated fatty acid; TCA: tricarboxylic acid cycle; TNF*α*: tumor necrosis factor *α*; IL-1: interleukin-1; IL-6, interleukin-6; SHBG: sex hormone binding globulin; E2: estradiol; IGF-1: insulin-like-growth-factor-1.

It is well established that chronic inflammation increases cancer risk [90]. Obesity is associated with adipose tissue inflammation, characterized by macrophage infiltration into the adipose deposit [12]. In addition, large adipose deposits in obese individuals could have limited blood supply resulting in adipose tissue hypoxia [91] and induction of hypoxic-inducible factor 1-*α* (HIF-*α*) expression in adipocytes. HIF1*α* in turn stimulates expression of monocyte chemotactic factor 1 (MCP1), promoting macrophage recruitment to adipose deposits. In addition, production of saturated fatty acids by the breakdown of large fat droplets in large adipocytes in obese individuals can lead to activation of caspase-1, interleukin-1 *β*, and the NF-*κ*B signaling cascade [92]. Together, adipocytes and macrophages in adipose tissue induce expression of inflammatory cytokines such as TNF-*α*, IL-6, and prostaglandin-E2 (PGE2) [90] (Figure 1).

Both TNF*α* and PGE2 have been shown to stimulate expression of aromatase in adipose tissues and increase the risk of breast cancer, especially in obese postmenopausal women [93, 94]. Expression of aromatase in fibroblasts or stromal cells surrounding the adipocytes and mammary ducts helps to convert circulating androgen into estrogens in the breast. Examination of aromatase activity and expression of aromatase in breast tissues from different quadrants surrounding breast tumors found that aromatase activity and expression were highest in the quadrant that contained the tumor and decreased with increasing distance away from the tumor [95, 96]. The increase in the aromatase expression was shown to be largely due to the levels of PGE2 produced by the tumor, suggesting that inflammatory cytokines were produced by the adipocytes and tumors of obese women [96]. TNF*α* via NF*κ*B signaling stimulates aromatase expression through CYP19A1 (aromatase) promoter I.4 by stimulating the binding of c-Fos and c-Jun transcription factors to the activation protein-1 (AP-1) element upstream of promoter I.4, while PGE2 stimulates aromatase expression through promoter II/I.3 of the human CYP19A1 gene [13]. Recent studies showed the presence of crown-like structures, macrophages surrounding large lipid-filled adipocytes of obese subjects, in the breasts of obese women [97]. The presence of these crown-like structures correlated with an increase in NF*κ*B expression and elevated aromatase activity [97]. Further study indicated that the increase in aromatase expression correlated with an increase in the activity of the CYP19A1 proximal II/I.3 promoter and is associated with an increase in the cyclooxygenase-2 (COX2) expression and PGE2 levels in the breasts of obese women [93].

### 6.1. Molecular Mechanism of ER and PR Crosstalk with NF*κ*B Inflammatory Signaling Pathway

The NF*κ*B pathways play crucial roles in the inflammation process in several tissues including mammary adipose tissues [98]. The influence of ovarian sex steroids on NF*κ*B signaling has been well documented. Both ER and PR have been shown to regulate cellular inflammation through interacting with NF*κ*B signaling pathways [98]. Constitutive activation of NF*κ*B signaling promotes the development of hormone-independent breast tumors [99]. Obese postmenopausal women have an increased risk for developing steroid hormone receptor positive breast cancer [100]. NF*κ*B activation in ER+ postmenopausal breast cancer is associated with endocrine resistance and a more aggressive phenotype [101]. Studies have demonstrated that ER interacts with RelA/p65, a member of the NF*κ*B family, and synergistically regulates expression of a group of genes that are important in regulating breast cancer cell survival and chemoresistance. ER interacts with RelA and enhances binding of both ER and RelA to ERE, resulting in activation of prostaglandin-E synthase-1 (PGES) gene expression leading to an increase in PGE2 production [102]. High levels of PGE promote breast cancer invasiveness, angiogenesis, and expression of aromatase in stromal and adipose tissue in the breast [103, 104]. In addition, ER can crosstalk with the NF*κ*B pathway through interaction between ER and I*κ*B Kinase *α* (I*κΚα*) at promoters of ER target genes, important for breast cancer cell-cycle progression including cyclin-D1 and E2F1 [105, 106]. Together these studies demonstrate that obesity could promote inflammation and the development of more aggressive, invasive breast cancer phenotypes and chemo- and endocrine resistant breast cancers.

In some cases, ER has been shown to repress NF*κ*B activity and may be involved in anti-inflammatory effects of E2. ER inhibits NF*κ*B signaling through NF*κ*B DNA binding or NF*κ*B transcriptional activation [107]. ER represses expression of RelB, a member of the NF*κ*B family, by directly or indirectly preventing NF*κ*B and AP-1 interaction at the RelB promoter, resulting in formation of an unfavorable DNA complex for transcriptional activation [108]. In addition, a study by Nettles and colleagues suggested that transrepression of NF*κ*B activity by ER may occur through a competition between ER and NF*κ*B, displacing cAMP response element-binding protein-binding protein (CBP)/p300 coactivator from the promoter of NF*κ*B target genes and resulting in suppression of NF*κ*B target genes [109].

Increasing evidence suggests that PR plays a significant role in anti-inflammatory responses in breast cancer cells [110, 111]. Interestingly, lack of PR in ER+ breast cancer is associated with less differentiated, more aggressive breast tumors that are resistant to endocrine therapies [112]. A recent study demonstrated that PR through progestin-dependent and independent mechanisms decreased expression of aromatase, COX-2, and HER-2/neu [111]. PR expression or progesterone treatment was shown to increase the expression of the nuclear factor-*κ*B inhibitor (I*κ*B*α*). Expression of I*κ*B*α* positively correlated with PR expression in 28 breast cancer cell lines [110]. Gene expression analysis in both normal mammary epithelial and breast cancer cells revealed that PR inhibited NF*κ*B target genes through interacting with DNA-bound RelA/p65, resulting in transcription inhibition of NF*κ*B target genes. PR inhibited transcription of NF-*κ*B target genes through two different mechanisms: activation function-2 (AF-2) dependent and AF-2 independent [111]. Agonist-bound PR repressed the AF-2 dependent class of genes while the AF-2 independent class of genes was equally sensitive to both PR agonists and antagonists. Together, these studies suggested that the anti-inflammatory effect of PR is likely mediated through multiple mechanisms. PR agonists may inhibit some NF*κ*B target genes but may not affect others. More studies will be needed to exploit the anti-inflammatory role of PR and to design specific PR ligands to selectively inhibit NF*κ*B target genes that are crucial for the development and progression of breast cancer. Collectively, these data suggest an association between ER PR expression and obesity induced inflammation. Chronic inflammation induced by adipocytes and macrophages in adipose tissue could be further modulated by ER or PR signaling. Thus, interfering with ER PR signaling may help reduce obesity induced inflammation and decrease the incidence of breast cancer in obese postmenopausal women.

### 6.2. Possible Role of ER, PR in Obesity-Induced Inflammation and Postmenopausal Breast Cancer: Clinical Evidences and* In Vivo* Models

Systemic analyses of clinical studies have suggested that obese postmenopausal women have almost a twofold increase in hormone receptor positive (ER+/PR+) breast cancer [113]. Data from several* in vitro* and* in vivo* studies indicated that chronic inflammation induced by obesity causes an increase in inflammatory mediators, leading to an increase in aromatase activity and expression in adipocytes and hormone-dependent breast cancer. Extragonadal local E2 production, induced by chronic inflammation, in the breast of obese postmenopausal women supplies breast epithelial cells with E2 and increases the risk for breast cancer by promoting the growth of hormone receptor positive breast cancer in these women.

*In vivo* models of diet-induced obese animals have provided further support for obesity and development of breast cancer in postmenopausal women. In a recent study in a diet-induced obesity model, Wistar rats were fed with a high fat diet and were classified into three categories: obese, mid-weight, and lean [21]. Mammary tumors were induced by a single injection of 1-methyl-1-nitrourea (MNU) into rats at about age 52 days. MNU-induced rat mammary tumors are similar to breast tumors seen in human breasts in term of percentage of intraductal tumors and pattern of progression and steroid receptor positivity [114]. Rats in the obese group were allowed to gain weight under the obesogenic conditions of high-fat diet and were restricted to limited physical activity; and some rats were also ovariectomized (OVX) at 19 weeks of age (obese-OVX). Tumor numbers were similar in all groups before OVX. After OVX, obese-OVX group showed almost a twofold increase in tumor numbers as compared to pre-OVX values while tumor numbers remained relatively the same in lean-OVX and mid-weight OVX-rats. Seventy percent (70%) of tumors in obese-OVX group expressed high ER*α* levels as compared to 30% of mammary tumors from non-obese-OVX rats, similar to breast tumors seen in obese postmenopausal women with a high portion of tumors classified as receptor-positive. It is likely that extragonadal local E2 production in adipose tissue of OVX-obese animals fostered the development of ER+ tumors.

An association of obesity and progesterone receptor positivity in mammary tumors has recently been demonstrated in an* in vivo* obesity and overfeeding rat model conducted in the MacLean laboratory [20]. Rats were classified into four groups, including obesity-prone (obese) and obesity-resistant (lean) groups and heaviest and lightest groups. These groups were further subdivided into low and high energy excess group based on energy expenditure within the last 48 hour prior to sacrifice. Mammary tumors from obese animals with high-energy excess had statistically significantly higher glucose uptake than that of tumors from obese with low-energy excess and lean rats. While lean animals preferred to deposit excess nutrients in mammary and peripheral tissues, obese animals deposited their excess nutrients into their mammary tumors. Interestingly, tumors from obese animals showed an increase in PR expression, mainly the PR-A isoform in preference to the PR-B isoform [20]. Furthermore, elevated PR expression was positively correlated with expression of glycolytic and lipogenic enzymes, glucose uptake, and proliferation markers in mammary tumors. Treatment with metformin, an antidiabetic drug, during OVX-induced obesity resulted in tumor regression and a decrease in PR expression [20]. Consistent with* in vivo* findings, analysis of expression microarrays of breast tumors from postmenopausal women revealed that PR+ tumors had an increase in metabolic enzymes similar to that of PR+ tumors from the obese-prone rat model, suggesting that PR+ breast tumors have increased metabolic activities. Interestingly, PR-A has been shown to induce inflammatory processes in the mammary gland by promoting leukocyte recruitment to mammary epithelium [115]. Data obtained from these animal models are consistent with increased risk of developing hormone receptor positive breast cancer among obese postmenopausal women [7]. These studies also suggested that expression of PR-A in breast tumors enhanced metabolic capacity and induced proinflammatory responses in breast cancer. Most clinical studies reported PR positivity in breast tumor specimens without isoform specification. Therefore, future studies will be needed to determine the role of PR isoforms in breast cancer metabolism and aggressiveness.

## 7. Conclusion and Future Perspectives

How the cessation of ovarian functions and withdrawal of ovarian steroids, E2 and progesterone, promote postmenopausal obesity and contribute to the development of hormone receptor positive breast cancer is summarized in Figure 1. The lack of estrogen and progesterone after menopause can cause an imbalance in a woman's homeostasis. The lack of estrogen can bring about a decrease in insulin sensitivity and glucose uptake and a decrease in glucose metabolism and mitochondria respiration. The lack of ovarian steroids, especially estrogen, could lead to a reduction in cellular metabolism and a reduction in total energy expenditure. Reduction in energy expenditure together with lack of physical activities and high fat diet promote postmenopausal weight gain and obesity [116]. Large fat deposits in obese women could limit blood and oxygen supply to the area and can result in tissue hypoxia. A hypoxic state induces expression of HIF1*α* and inflammation by promoting macrophage recruitment and secretion of inflammatory cytokines such as IL-6, IL-1*β*, PGE2, and TNF*α*. In addition, induction of HIF1*α* may also cause a shift in cellular metabolism to favor aerobic glycolysis often found in actively proliferating or cancer cells. The breakdown of large lipid droplets in obese women could cause an increase in saturated fatty acid (FAS) production and activation of inflammatory signaling pathways such as NF*κ*B activation. The release of inflammatory cytokines and activation of the NF*κ*B pathway help activate the CYP19A1 promoter and increase aromatase gene expression. Aromatase expression in the adipose tissue helps produce extragonadal estrogen from androgen in the surrounding tissues. Studies, as described in this review, suggest that obese women have lower levels of SHBG. Local extragonadal estrogen production together with low SHBG makes estrogen readily available to enhance the growth of ER+ breast cancer cells. Furthermore, obesity and the lack of ovarian sex steroids could help promote an insulin-resistant state with high circulating insulin and IGF-I. Leptin production from adipocytes is increased in obese individuals and increasing evidence shows a positive correlation between increasing leptin levels and the risk for postmenopausal breast cancer [117]. High levels of circulating IGF-1, insulin, and leptin may help promote the development and growth of breast cancer cells in postmenopausal women. Together, these data demonstrate a link between ovarian sex steroids, obesity, and inflammation with postmenopausal breast cancer.

While several advances have been made in our understanding of the role of ovarian steroids and their receptors on postmenopausal breast cancer, many important factors remain unclear and will need to be further explored to fully exploit their possible beneficial effects after menopause. Studies suggested that AF2 of ER*α* is required to mediate E2 beneficial metabolic effects such as insulin sensitivity and energy expenditure, in an ERE-independent mechanism [68]. However, a detailed molecular mechanism of how ER*α*-AF2 is involved in these effects remains unclear. Better understanding of this molecular mechanism could help us develop a specific ligand that could specifically harness beneficial metabolic effects of ER without its harmful side effects.

Leptin plays a significant role in breast cancer development and progression [117]. Leptin stimulates production of proinflammatory cytokines and promotes cell proliferation and angiogenesis in breast cancer [117]. E2/ER stimulates leptin production both in human subjects and in* in vivo* animal models [118]. However, a detailed molecular mechanism of how E2 induces leptin expression and promotes leptin sensitivity is still to be explored. Better understanding of how E2/ER increases leptin levels could help devise new strategies for breast cancer prevention and reduce breast cancer incidence in obese postmenopausal patients.

How progesterone/PR affects cellular metabolism remains unclear. However, a recent study in an overfeeding OVX obese rat model for postmenopausal obesity showed that mammary tumors from obese rats with high energy excess overexpressed PR, mostly in form of the PR-A isoform [20]. The association between high PR expression and obesity or overfeeding is an interesting area of study. PR+ tumors showed an almost 50% increase in glucose uptake and lower retention of dietary fat as compared to PR− tumors [20]. Whether PR directly or indirectly influences cellular metabolism or whether PR-B could exert similar metabolic effects in breast cancer is under investigation. Future studies will be required to unravel the association between high PR expression and glucose metabolism in breast cancer cells. In addition, it will be interesting to examine how expression of different PR isoforms affects breast cancer cell metabolism.

Progesterone/PR has an anti-inflammatory role in several animal models and in breast cancer cells [110, 111, 119]. Since obesity can induce a state of chronic inflammation [13], it would be interesting if we could take advantage of PR's anti-inflammatory function to reduce inflammation caused by obesity. Additional studies will be necessary to determine detailed molecular mechanisms of how PR exerts its anti-inflammatory function and to enable the design of specific PR ligands that specifically activate PR's anti-inflammatory activity. Metformin, the antidiabetic drug, has been shown to decrease PR expression and induce regression of mammary tumors and endometrial cancer [20, 120]. While a recent study suggested that metformin promotes PR expression by increasing AMP-activated protein kinase (AMPK) phosphorylation and by inhibiting the mTOR pathway [120]. Future studies will be needed to determine the molecular mechanism of how metformin affects breast cancer growth and PR expression.

Depletion of ovarian sex steroids, E2 and progesterone, in combination with limited physical activity and high fat diet after menopause could cause several metabolic changes that facilitate weight gain and obesity. Obesity in E2 and progesterone deprived states in postmenopausal women may cause imbalance in homeostasis and changes in epithelial cells, stromal cells and adipocytes in the breasts that promote the growth and survival of receptor positive breast cancer cells (Figure 1). Better understanding of molecular mechanisms and signaling pathways that mediate these effects will help us to design better cancer prevention and treatment strategies for postmenopausal breast cancer.
